# Impact of Slowly Biodegradable COD and Loosely Bound Polymeric Substances Accumulation in High-Rate Activated Sludge: Implications for Bioflocculation and Organic Matter Harvesting

**DOI:** 10.1021/acsestengg.5c00745

**Published:** 2025-10-01

**Authors:** Zoé Fau, Antonin Azais, Sylvie Gillot, Florent Chazarenc, Nicolas Derlon

**Affiliations:** † INRAE, UR REVERSAAL, 5 rue de la Doua, 69625 Villeurbanne, France; ‡ Swiss Federal Institute of Aquatic Science and Technology, 28499 Eawag, Ueberlandstrasse 133, 8600 Dübendorf, Switzerland

**Keywords:** bioflocculation, biopolymer, loosely and tightly bound polymeric substances, high-rate activated sludge, organic matter harvesting

## Abstract

This study investigates the impact of loosely bound (LB-) and tightly bound (TB-) polymeric substances (PS) on bioflocculation and organic matter harvesting in High Rate Activated Sludge (HRAS) systems, operated with primary effluent wastewater to specifically investigate the bioflocculation process. A pilot-scale HRAS system was operated at a contrasted solids residence time (SRT) of 0.2 and 0.8 d to assess the composition of polymeric substances extracted from the sludge (LB- vs TB-contents, biopolymers fraction), bioflocculation capacity, settleability, and the fate of organic matter. Results demonstrate that a low SRT (0.2 d) favors the accumulation of influent slowly biodegradable COD (more than 60% based on COD mass balance) and of LB-PS with a limited biopolymer content (<30%). The high LB-PS content observed at 0.2 d SRT (259 ± 15 mgCOD/gVSS) in turn hinders bioflocculation, resulting in the formation of small and loose flocs and a higher loss of effluent suspended solids. Conversely, sludge grown at 0.8 d SRT exhibited a lower LB-EPS (116 ± 9 mgCOD/gVSS) content with a better bioflocculation, resulting in the formation of larger, more structured and fluffier flocs. A poor bioflocculation at low SRT hampered particulate and colloidal organic matter removal, ultimately limiting the harvesting of organic matter despite an increased redirection. Overall, our results provide relevant insights into the role of sludge composition (influent slowly biodegradable COD, LB-PS, biopolymers content) in the bioflocculation and resulting harvesting of organics in HRAS systems. Our results also suggest that operation of HRAS systems at a very low SRT (e.g., 0.2 d) has the potential to increase the harvesting and valorisation of the organic matter of municipal wastewater but requires a better control of bioflocculation.

## Graphical abstract

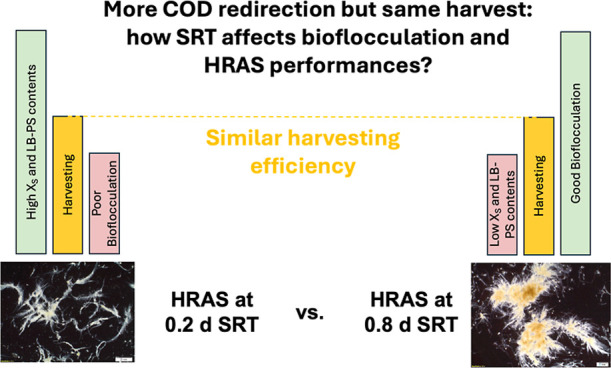

## 1 Introduction

Future wastewater treatment plants must address critical environmental and societal challenges such as minimizing their environmental footprints or promoting resource recovery, while maintaining their primary sanitation role. A relevant approach to address these challenges consists of maximizing the harvesting of organics present in wastewater (WW). Organics harvesting reduces energy demand in downstream processes while improving the production of biogas or other valuable products.^1^ High Rate Activated Sludge (HRAS) is an alternative to Conventional Activated Sludge (CAS) which requires less energy and maximizes organic matter recovery.^2^ Although HRAS systems have been implemented at a full scale for many years, the mechanisms governing organic capture, particularly bioflocculation, remain poorly understood, thus limiting the optimization and performance of these systems.

HRAS systems aim at maximizing biosorption through surface sorption and intracellular storage mechanisms (i.e., organics capture), while minimizing organic matter oxidation. Operating conditions such as very low Sludge Retention Time (SRT) (0.1–2.0 d), short Hydraulic Retention Time (HRT) (0.1–2.0 h), and low Dissolved Oxygen (DO) concentration (0.5–1.0 mgO_2_/L) are maintained to reduce oxidation and increase organic matter harvesting.^3,4^ However, low SRT and HRT conditions result in a poor effluent quality, as indicated by the high Effluent Suspended Solids (ESS) concentrations observed for HRAS systems: up to 4 times higher than in CAS.^5^ As a result, the harvesting efficiency of the HRAS system is hampered, thus reducing the net valorization of organic matter. A poor bioflocculation of the sludge has been proposed to be responsible for the elevated ESS concentrations.^6^ There is, however, little understanding of the factors that control bioflocculation and in turn on how to maximize the harvesting of organic matter. Improving the sludge bioflocculation to enhance the capture of small particles and colloids thus remains a major challenge in order to increase the harvesting efficiency of HRAS systems.^5^

Bioflocculation refers to the formation of flocs capable of settling under the condition imposed by the system.^5^ Collison rate, collision efficiency, and floc strength are the drivers of bioflocculation.^7^ The effect of collision rate on bioflocculation is rather well-understood and depends on the Mixed Liquor Suspended Solids (MLSS) concentration and shear intensity.^8^ In contrast, collision efficiency and floc strength are influenced by sludge properties, such as charge, size, and hydrophobicity, and remain less understood.^9,10^ EPS are a major constituent of bacterial aggregates, comprising 50 to 80% in mass of the organic fraction of activated sludge.^11^ Proteins and polysaccharides are the primary component of EPS, but EPS also contain nucleic acids and lipids and molecules that are not of microbial-origin such as humic substances.^12,13^ Due to their spatial organization, quantity, and composition, EPS play an essential role in the formation and mechanical stability of flocs.^10,13^ A common approach to distinguishing EPS is to classify them according to their binding to the cell, for example by differentiating between loosely bound and tightly bound EPS. Loosely bound (LB)-EPS are weakly attached and therefore easily extracted, while tightly bound (TB)-EPS are more strongly attached to the cell.^13^ But despite their importance, the effect of EPS on floc formation and bioflocculation is poorly understood.^4^ EPS are more and more studied, but results are contrasting, whether in the case of CAS^14,15^ or HRAS.^7,16–19^ Initial results suggest that EPS composition is more influential of bioflocculation than total concentration.^5,20^ A high ratio of protein/polysaccharides has been reported to favor bioflocculation, but a clear consensus on the impact of EPS composition on bioflocculation is still missing.^7,14,17^ Also, limited attention has been given to TB- and LB-EPS. Contradicting conclusions have been reported on the role of TB- and LB-EPS in bioflocculation or settling in activated sludge processes. Links between bioflocculation and settling are in most of the studies made with LB-EPS but rarely with TB-EPS, which seem to have a lesser impact.^21,22^ LB-EPS are suspected to limit bioflocculation and settling in most studies on CAS^21–23^ or HRAS.^7,8,17,24^ However, LB-EPS are sometimes associated with good bioflocculation capacity.^25^ Finally, it is important to highlight that the selected EPS extraction method has a strong influence on the EPS content and composition that is measured. Also, as opposed to conventional activated sludge, sludge from the HRAS system is enriched with particulate matter from the influent, which may undergo partial solubilization during EPS extraction, potentially influencing the results. Then, the content and composition of the LB- and TB-“EPS” extracts could, in theory, be influenced by the solubilization of particulate matter originating from the influent. Therefore, to account for the potential influence of influent-derived particulate matter solubilization on the extracted fractions, we will use the term polymeric substances (PS) rather than extracellular polymeric substances (EPS) throughout this manuscript. Further investigation on the impact of SRT on TB- and LB-PS composition and its role in bioflocculation is, however, required for optimizing HRAS processes.

The main objective of this work is therefore to better understand how EPS composition in terms of TB- and LB-EPS content and composition affects the bioflocculation of sludge and in turn the harvesting efficiency of the HRAS system, i.e., how much organics are captured and then valorised. The specific research questions were addressed: (i) how does SRT influence the fate of organic matters (particulate, colloidal, and soluble fractions) and in turn its redirection into biomass and its harvesting, (ii) how are the differences in the harvesting of organics influenced by differences in the bioflocculation and settling of the sludge, and (iii) how can sludge content and biopolymers content of TB- and LB-EPS explain the bioflocculation capacity of the sludge. A HRAS reactor was operated at a distinct SRT of 0.2 and 0.8 d SRT to address those questions. The fate of organic matter, the bioflocculation and settling properties of the sludge (ESS, SVI_30_, Threshold Of Flocculation), and the biochemical composition of the sludge (TB- and LB-EPS sludge content and biopolymers content in TB- and LB-EPS) were monitored for each SRT condition. Data from our study were analyzed together with literature data, selected to ensure comparability in operating conditions (operation under contrasted SRT conditions in a range of 0–1 d) and in the type of parameters reported. Only studies providing relevant metrics, such as COD fractionation, settling properties, and EPS quantification, were included in this comparison and further discussed.

## 2 Materials and Methods

### 2.1 Experimental Approach

A pilot-scale HRAS was operated at two distinct SRTs of 0.2 and 0.8 days and fed with primary effluent wastewater. Primary effluent wastewater was used in our study to ensure that settling and carbon capture in the HRAS are primarily governed by bioflocculation, as settleable solids present in the raw WW are mostly removed in the primary clarifier. Two different SRT conditions were applied to trigger distinct behaviors in terms of EPS composition, bioflocculation, and settling properties and ultimately in terms of harvesting efficiency (Table 1). Values of 0.2 and 0.8 d SRT were selected based on previous results from Jimenez et al.^16^ who reported that specific EPS concentration doubled and particulate (pCOD) and colloidal (cCOD) Chemical Oxygen Demand removal rates tripled when SRT increased from 0.3 to 1.0 d. A 2 h HRT, a DO set-point of 0.5 mgO_2_/L on average, and a temperature ranging between 19 and 24 °C were maintained. For each measurement, a MLSS grab sample was collected from the bioreactor and analyzed for COD fractions, Total Suspended Solids (TSS), Volatile Suspended Solid (VSS), bioflocculation, settling, and EPS within 3 h after sampling. 24 h composite samples of the influent and the effluent were collected using a refrigerated autosampler and directly analyzed for TSS, VSS, and COD fractions.

**Table 1:** Experimental Conditions Maintained during Operation at 0.2 and 0.8 d SRT

	SRT 0.2 d (*n* = 4)	SRT 0.8 d (*n* = 3)
aerobic SRT (d)	0.2 ± 0.1	0.8 ± 0.2
total SRT (d)	0.4 ± 0.1	1.1 ± 0.2
HRT (h)	2	2
Dissolved Oxygen (mgO_2_/L)	0.5 ± 0.5	0.5 ± 0.3
MLSS (gTSS/L)	0.4 ± 0.1	1.3 ± 0.3
reactor temperature (°C)	19–24	19–24

For each SRT condition, only data corresponding to the steady-state phase under dry weather conditions (in terms of COD removal, effluent quality, SVI_30_, and EPS content) are reported and used for further calculations. Approximately 15 days were required to reach a steady state after changing the SRT. Only dry weather conditions were selected as the HRAS process was sensitive to wet weather due to the very short SRT. In total, four sampling days were selected for the 0.2 d SRT condition and three for the 0.8 d SRT condition. The aerobic SRT was calculated based on mass of solids in the HRAS and excess sludge/effluent suspended solids, thus neglecting the influent solids but enabling comparison with values provided in the literature (calculated based on similar assumptions). The total SRT was calculated considering the total mass of solids in the system (HRAS plus clarifier) and excess sludge and effluent suspended solids.

### 2.2 Experimental Setup and Influent Composition

The HRAS pilot consisted of a 564 L bioreactor connected to a 280 L cone bottom clarifier operated at a surface overflow rate of 0.7 m/h. The HRAS was fed with primary effluent wastewater at a flow rate of 282 L/h. SRT was based on the biomass inventory in the reactor and was controlled by the wasting of mixed liquor from the bioreactor. The recirculated flow rate was 80% of the influent flow. The DO concentration was measured using an optical sensor (Oxymax COS61D, Endress + Hauser) and maintained by proportional-integral-derivative (PID) control. Compressed air was injected into the system through membranes in the form of fines bubbles.

Average characteristics of the influent are summarized in Table 2. Influent composition was almost the same in terms of TSS and COD content at 0.2 and 0.8 d SRT conditions (less than around 20% of difference). The influent came from a primary clarifier operated at an HRT of 1–2 h resulting in a TSS removal of around 50% and a primary effluent therefore free of settleable particles.

**Table 2:** Average Influent Characteristics Measured on 24 h Samples

parameters	units	SRT 0.2 d (*n* = 4)	SRT 0.8 d (*n* = 3)
TSS	mgTSS/L	125 ± 8	102 ± 7
VSS/TSS		0.90 ± 0.01	0.88 ± 0.01
tCOD/TSS		2.9 ± 0.3	2.9 ± 0.3
tCOD	mgCOD/L	360 ± 38	291 ± 13
pCOD	mgCOD/L	233 ± 23	187 ± 11
cCOD	mgCOD/L	60 ± 6	56 ± 8
ffCOD	mgCOD/L	67 ± 20	48 ± 16

### 2.3 Analytical Methods

#### 2.3.1 Influent, Effluent, and Mixed Liquor Characteristics

Total Suspended Solid (TSS) and Volatile Suspended Solid (VSS) were determined with standard methods.^26^ COD was fractionated using the method developed by Mamais et al.^27^ to determine the pCOD (>1.5 μm), cCOD (difference between pCOD and ffCOD), and flocculated filtered COD (ffCOD) (<0.45 μm after flocculation) concentrations. COD concentration was measured using Hach Lange micromethods. For the mixed liquor COD measurement, samples were first homogenized by Ultraturax.

#### 2.3.2 Bioflocculation and Settling Properties Indicators

Effluent Suspended Solids and Sludge Volume Index after 30 min (SVI_30_) were measured according to the standards methods.^26^ Also, the Threshold Of Discrete Flocculation (TODF) and the Threshold Of Flocculation (TOF) were quantified to characterize the bioflocculation of the sludge.^28^ The TODF and TOF curves describe the relationship between supernatant TSS and the initial TSS concentration. The TODF is the lowest initial TSS concentration at which the supernatant TSS deviates from the initial TSS, indicating the start of sedimentation. The TOF is defined as the minimum initial TSS at which sedimentation accelerates due to pronounced bioflocculation, as evidenced by a clear break in the slope of the TODF or TOF curve. TODF is therefore indicative of the initial start of bioflocculation with minimal impact on sedimentation, whereas TOF indicates a more advanced stage, where flocculation is pronounced enough to markedly speed up sedimentation and reduce the supernatant TSS.

TODF and TOF experiments were performed in a Plexiglas column with a volume of 1.7 L, a diameter of 9 cm, and a sample port located at 5 cm below the surface. Samples were collected after 2 min of sedimentation, corresponding to a critical settling velocity of 1.5 m/h. Between 6 and 8 different dilutions of the sludge samples were tested. Diluted sludge was poured into the column with a funnel to avoid any vortexing that could alter bioflocculation. A 100 mL sample was collected to determine the initial TSS concentration. Sludge was allowed to settle for 2 min, prior to sampling the supernatant located above to the sampling port, thus corresponding to a settling velocity of 1.5 m/h. TODF and TOF values were determined using the numerical method proposed by Fau et al.^28^

#### 2.3.3 Sludge Morphology

Sludge morphology was observed using stereomicroscopic images obtained with an SZX10 Olympus stereomicroscope and a SC30 Olympus camera. The stereomicroscope allows the floc structure to remain unchanged as it is not necessary to place flocs between a slide and a cover slide. Fresh samples were observed without any concentration standardization, so images provided qualitative information only.

#### 2.3.4 Extraction of Loosely (LB) and Tightly Bound (TB) Polymeric Substances (PS)

LB- and TB-polymeric substances were extracted using a heat extraction modified method based on the work from Li and Yang.^22^ A volume of 1L of sludge was first centrifuged for 5 min at 3200*g* to remove the soluble EPS. The pellet was then resuspended to its initial volume of 1 L in a sodium chloride (NaCl) solution (0.05% w/v) preheated at 60 °C and centrifuged for 10 min at 3200*g*. The supernatant was collected as an LB-extract. The pellet was resuspended in NaCl solution, mixed at 200 rpm for 30 min at 60 °C, and centrifuged for 20 min at 3200*g*. The supernatant was collected as TB-extract. The temperature from the initial method was decreased from 70 to 60 °C to avoid cell lysis.^29^ This method enables one to extract separately LB- and TB-polymeric substances, to limit cell lysis due to the limited temperature increase, and to have a representative extract of polymeric substances and is easily performed on site. Once extracted, solubilized polymeric substances were quantified by measuring the total COD content using Hach-Lange micromethods and expressed in terms of mgCOD/gVSS.

Biochemical Composition of the Loosely (LB) and Tightly Bound Extracts (Liquid Chromatography Coupled with Organic Carbon and Organic Nitrogen Detectors (LC-OCD-OND))

The biochemical composition of the LB- and TB-extracts was analyzed using LC-OCD-OND, which separates and quantifies the dissolved organic matter into five fractions of different molecular weights (MW), charges, and chemical functions: biopolymers (MW > 20,000 Da), humic substances (MW ∼1000 Da), building blocks (MW ∼300–500 Da), low molecular weight (LMW) humics (MW < 350 Da), LMW acids (MW < 350 Da), and LMW neutrals (MW < 350 Da). A size exclusion chromatography (SEC) column consisting of 50–50% Toyopearl TSK HW50S and HW65S, respectively, was used for the separation of the different MW compounds. Calibration was performed using different molecular weight standard mixtures of pullulan (708 kDa–180 Da, PSS) and thyroglobulin (669 kDa), ferritin (440 kDa), alcoholdehydrogenase (150 kDa), conalbumin (75 kDa), and ovalbumin (44 kDa). The quantification limit of the method was 50 μgC/L. The protein content (MW > 20,000) of the solubilized EPS was approximated via the quantification of the nitrogen to carbon ratio in the extract (mgN in the biopolymer fraction/mgC in the hydrophilic fraction).

### 2.4 Calculations

#### 2.4.1 COD Removals and Fate of COD (Redirection, Harvesting, and Oxidation)

Process performances in terms of pCOD, cCOD, and ffCOD and fate of organic matter (i.e., redirection into biomass, harvesting through excess sludge, or oxidation) were quantified. Measurements were performed on 24 h composite samples. COD removal rates and the fate of COD were calculated using the method introduced by Rahman et al. (2019).^6^ The fate of COD is analyzed based on the calculation of the fraction (%) of the influent COD (COD_in_) that leaves the reactor in the effluent (COD_eff_) and in the waste activated sludge (COD_was_) and that is oxidized (COD_ox_). COD_ox_ is calculated by subtracting COD_eff_ and COD_was_ from COD_in_. Then organic matter redirected into biomass (COD_red_) and harvested (COD_harv_) and harvesting efficiency can be calculated.

1 $${COD}_{red}={COD}_{was}+{pCOD}_{eff}+{cCOD}_{eff}$$

2 $${COD}_{harv}={COD}_{was}$$

3 $$harvesting\;efficiency=\frac{{COD}_{was}}{{COD}_{red}}$$

COD_red_ is the fraction of organic matter that converts into biomass through conversion and enmeshment. The “biomass” refers to active biomass, EPS, influent slowly biodegradable substrate, etc. COD_red_ is the sum of the percentage of COD_was_, pCOD_eff_, and cCOD_eff_ relative to the COD_in_. The fraction of COD that is harvested represents the fraction of organics that can be recovered by the HRAS system and is equal to that of COD_was_. Harvesting efficiency, equal to the percentage of organic matter harvested compared to that redirected, is measured as the ratio between COD_was_ and COD_red_.

During the 0.8 day SRT condition, additional TSS losses in the effluent have been observed on an occasional basis, due to a hydraulic issue in the process leading to an overflow. The additional TSS losses in the effluent were therefore not representative of the physico-biological functioning of the process and distorted the 24 h composite samples by overestimating the pCOD. Therefore, pCOD in 24 h composite samples was calculated using the pCOD_was_/TSS_was_ ratio and the ESS concentrations measured during periods without overflow.

A systematic statistical analysis was performed on our individual data sets (at SRT of 0.2 vs 0.8 d) and on mean values from both our study and literature. Normality of the data distribution was first tested. Welch’s *t*-test or the Wilcoxon test was then performed, depending on the normality test results, to statistically compared data at 0.2 vs 0.8 d SRT. A linear model was applied to test the relationship between the SRT and observed variables. Such analyses were performed for several observed variables: removal rates (pCOD, cCOD, and ffCOD), COD_red_ and COD_harv_, LB-PS, TB-PS, and ratio of TB/LB. Normality of residuals was further tested when a linear model was applied.

#### 2.4.2 Estimation of the Slowly Biodegradable Substrate Concentration

Mass balance analysis on the slowly biodegradable substrate (X_S_) was conducted (Supporting Information SI1) to estimate the X_S_ concentration under the two experimentally tested SRT conditions (0.2 and 0.8 days). The results of EPS extraction, including the content and composition of LB- and TB-extracts, were then interpreted in relation to X_S_ accumulation in the sludge, as a function of SRT.

## 3 Results and Discussion

### 3.1 How Does SRT Influence the Fate of Organic Matter in HRAS?

#### 3.1.1 Effect of SRT on the Removal of the Particulate, Colloidal, and Soluble COD Fractions

The impact of SRT on the removal of the different COD fractions (particulate, colloidal, and soluble) was assessed at 0.2 and 0.8 days SRT and compared to results from the literature obtained with similar SRT (Figure 1). Our results indicate that pCOD, cCOD, and ffCOD removal rates increased with a SRT increase from 0.2 to 0.8 d. The pCOD removal was the highest and increased from 48 ± 6% (*n* = 4) at 0.2 d SRT to 84 ± 5% (*n* = 3) at 0.8 d SRT (Welch’s *t*-test, *p*-value = 0.0007). cCOD and ffCOD removal rates were lower than the pCOD removal rates but also increase with SRT: from 33 ± 16% (*n* = 4) to 57 ± 14% (*n* = 3) (Welch’s *t*-test, *p* = 0.084) and from 34 ± 19% (*n* = 4) to 58 ± 13% (*n* = 3) (Welch’s *t*-test, *p* = 0.105). However, these differences were not statistically significant.

**Figure 1:**
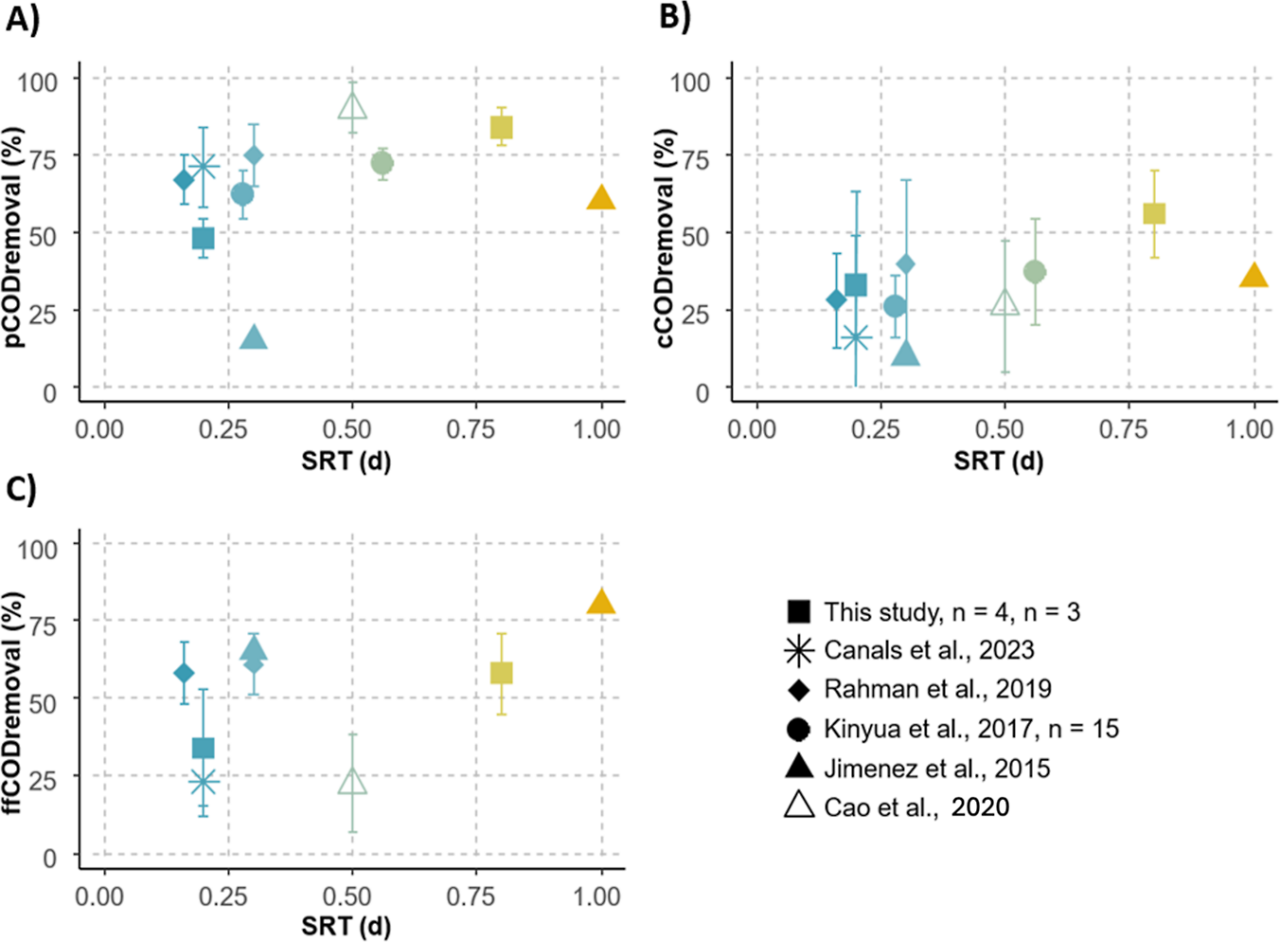
Change in the removal of pCOD, cCOD, and ffCOD as a function of SRT. Data presented are from this study and the literature. Bars represent standard deviation (not available for Jimenez et al.^16^) and “*n*” the number of measurements. Raw wastewater is the influent in all studies except ours.

When analyzing mean values from our study and from the literature, the apparent increase in pCOD, cCOD, and ffCOD removal rates with SRT was less pronounced (Figure 1). At SRT values of 0.2–0.5 d, pCOD removal rates ranged from approximately 50% to 75%, and increased slightly beyond 75% when the SRT exceeded 0.5 d. Likewise, cCOD and ffCOD removal rates seemed to increase from about 20% to over 50% and from about 25% to over 70%, respectively. However, statistical analysis of these mean values did not confirm a significant relationship between removal rates and SRT, which can be due to the variability of the operating conditions and WW composition of the different studies as well as the use of mean values for the statistical analysis.

#### 3.1.2 Effect of SRT on the Fate of COD in HRAS Systems

The fate of organic matter (among effluent, WAS, and oxidation process), its redirection into biomass, and ultimately its harvesting in a HRAS system fed with primary effluent WW, was monitored at an SRT of 0.2 and 0.8 days (Figure 2). COD_eff_, COD_was_, and COD_ox_ were not impacted similarly by SRT of the HRAS. COD_eff_ and COD_was_ decreased with an increasing SRT, from 56 ± 3 (*n* = 4) to 25 ± 3% (*n* = 3) and from 32 ± 6 (*n* = 4) to 10 ± 1% (*n* = 3), respectively, while COD_ox_ increased significantly from 11 ± 7 (*n* = 4) to 65 ± 2% (*n* = 3) when SRT increased from 0.2 to 0.8 d (Figure 2A–C).

**Figure 2:**
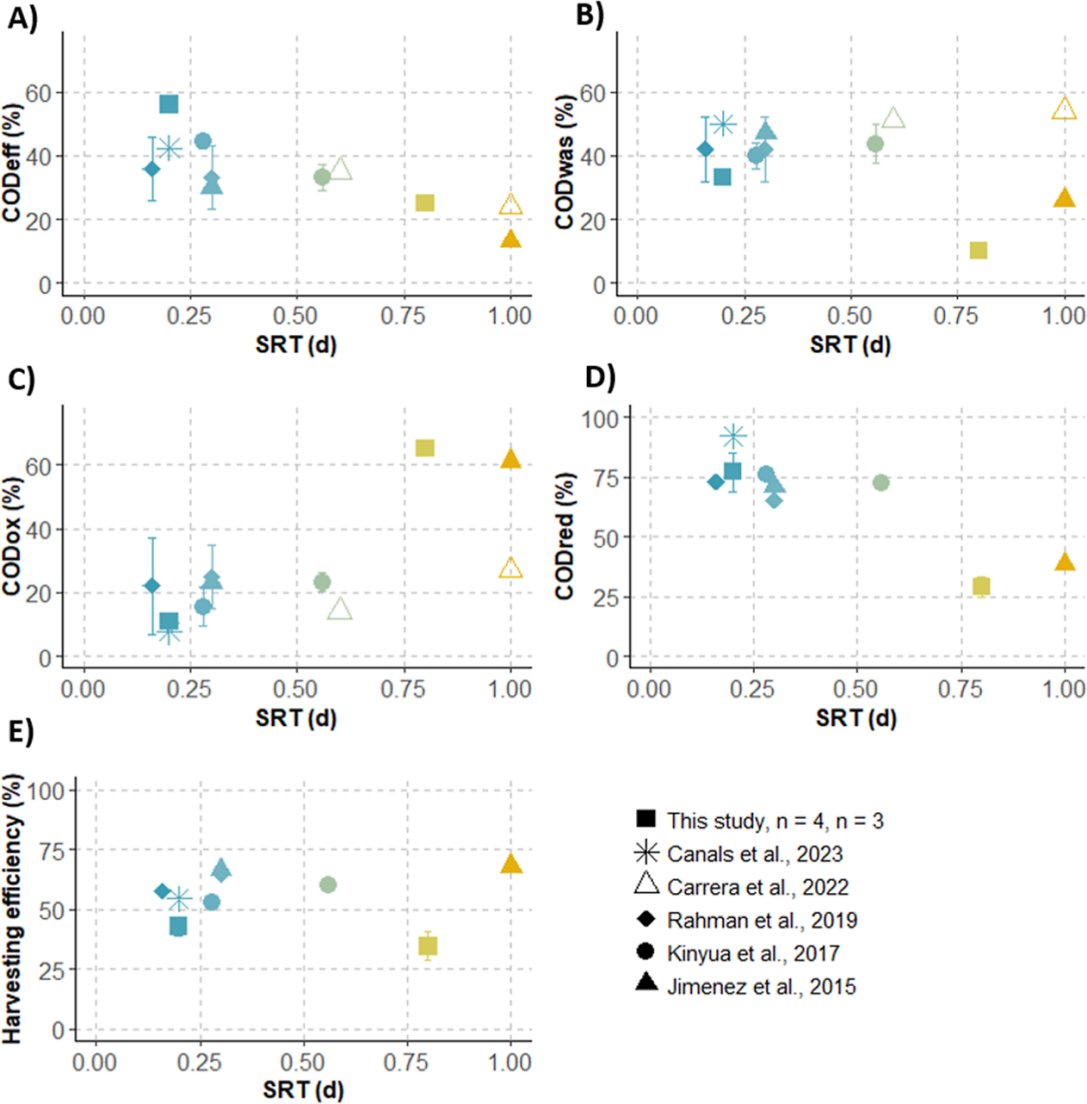
Change in the fractions of COD_eff_, COD_was_, COD_ox_, COD_red_, and harvesting efficiency as a function of SRT. Data presented are from this study and the literature. COD_red_ is the sum of the COD_was_ and particulate and colloidal COD_eff_. Harvesting efficiency is the ratio COD_was_/COD_red_. Bars represent standard deviations (not available for Canals et al.,^31^ Carrera et al.,^30^ and Jimenez et al.^16^) and “*n*” the number of measurements. For some points, standard deviations were too small to be visible on the plot.

Results from the literature are in accordance with our observations for COD_eff_ and COD_ox_. COD_eff_ decreased linearly from 50% to 20%, while COD_ox_ increased from 10% to 60% when SRT increased from 0.16 and 1.0 d SRT. The change in the COD_was_ with an increasing SRT was on the other hand quite variable among the different studies (Figure 2B). Jimenez et al.^16^ reported higher COD_was_ at low SRTs, consistent with our observations, although they found a smaller variation of COD_was_ with SRT because they used pre-screened raw wastewater, whereas our study was based on primary effluent. But Carrera et al.,^30^ Kinyua et al.,^17^ and Rahman et al.^19^ reported on the contrary on a slight increase from 43% to 54% of COD_was_ when SRT increased from 0.16 and 1.0 d.

The fate of COD among the effluent, WAS, and oxidation ultimately determines the fraction of COD that is redirected into biomass (COD_red_, Figure 2D). Our results indicate that COD_red_ decreased from 77 ± 8 (*n* = 4) to 28 ± 4% (*n* = 3) when SRT was increased from 0.2 to 0.8. Such decrease of COD_red_ with an increasing SRT is in line with results from the literature (decrease from 80 to 30% for a SRT increase from 0.16 to 1.0 d). A linear regression performed with the mean COD_red_ values from our study and literature suggested a strong negative correlation between COD_red_ and SRT (*p*-value = 0.003).

Harvesting efficiency is defined as the ratio between the COD_was_ and the COD_red_ and therefore represents a relevant indicator of the bioflocculation capacity of a sludge. Harvesting efficiency was calculated at 0.2 and 0.8 days of SRT and compared to results from the literature (Figure 2E). Both our results and results from the literature indicate a partial and constant harvesting efficiency of around 50%, which is maintained when SRT is varied in a range from 0.16 to 1.0 d SRT (*p*-value = 0.97). In our study, harvesting efficiency remained rather stable when SRT increased from 0.2 to 0.8 d, with low values from 42 ± 4 (*n* = 4) to 35 ± 6% (*n* = 3). Similar results were reported from the literature for SRT between 0.16 and 1.0 days, even if as for COD_was_ slight variability from one study to another can be observed. Overall, those results demonstrate that harvesting of COD does not increase proportionally to the redirection of COD into biomass when decreasing the SRT, due to the increase of COD loss via the effluent.

### 3.2 How Does SRT Influence Bioflocculation and Settling Properties?

Our previous observations demonstrate that SRT governs the performances of HRAS systems, e.g., the fraction of influent COD redirected into biomass (COD_red_) and ultimately to valorisation (COD_was_). A main question is now to understand to what extent those performances are governed by the bioflocculation and settling properties of the sludge. Our results, based on visual observations of flocs morphology and measurements of various indicators (ESS, harvesting efficiency, etc.), consistently indicated that bioflocculation/settleability is hampered at a low SRT of 0.2 d while improved at 0.8 d SRT.

#### 3.2.1 Floc Morphology (Stereomicroscopy)

Stereomicroscopic images were recorded at 0.2 and 0.8 days of SRT to provide qualitative information about the floc morphology in relation to SRT (Figure 3) (additional images available in Figure S1). Those visual observations indicate that floc morphology is markedly impacted by SRT. At 0.2 d of SRT, flocs were heterogeneous and characterized by filamentous structures (Figure 3A). Larger flocs were observed at 0.8 d SRT with a size of few millimeters and a very hairy morphology (Figure 3B). Flocs grown at 0.8 d SRT also seemed denser, as indicated by the darker color.

**Figure 3:**
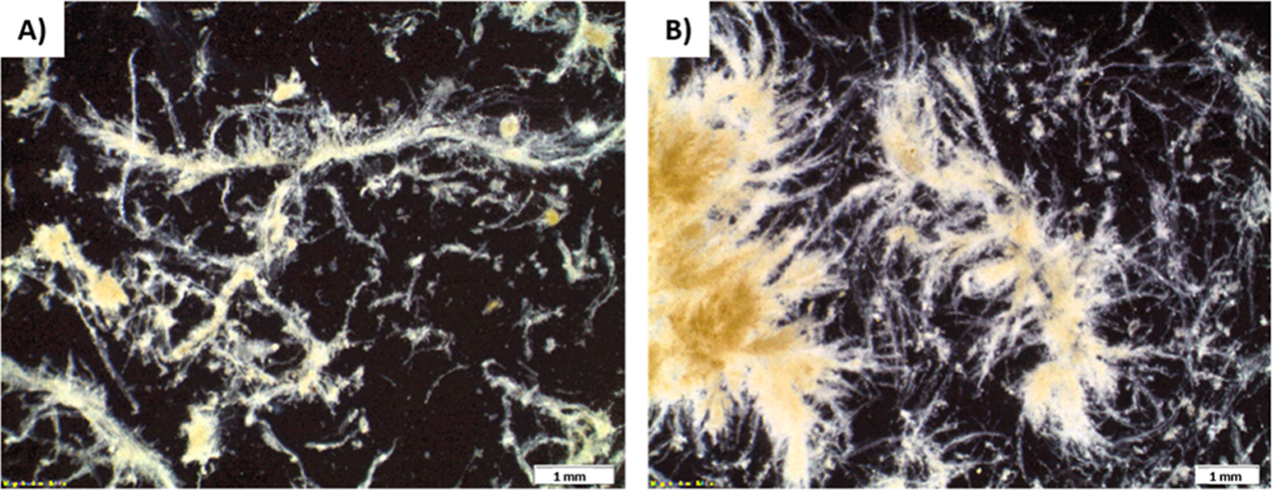
Stereomicroscopic images of flocs grown at (A) 0.2 d and (B) 0.8 d SRT.

#### 3.2.2 Effluent Suspended Solids (ESS) Concentration

Change in the ESS concentration as a function of SRT was measured and confronted to data from the literature (Figure 4). In our study, ESS concentration decreased strongly with an increase in SRT (Figure 4, square markers): from 83 ± 5 mg/L (*n* = 4) to 21 ± 7 mg/L (*n* = 3) as SRT increased from 0.2 to 0.8 d (−75% decrease). Overall, data from the literature indicate that ESS concentration decreased linearly with an SRT increase from 0.16 to 1.0 d. Similarly to our results, a variation of SRT from 0.16 to 1.0 d resulted in a significant reduction of the ESS concentration from 75 mg/L to below 20 mg/L. At a similar SRT, the ESS concentration is also influenced by the secondary clarifier design as reported by Canals et al.^32^ who tested different surface overflow rates.

**Figure 4:**
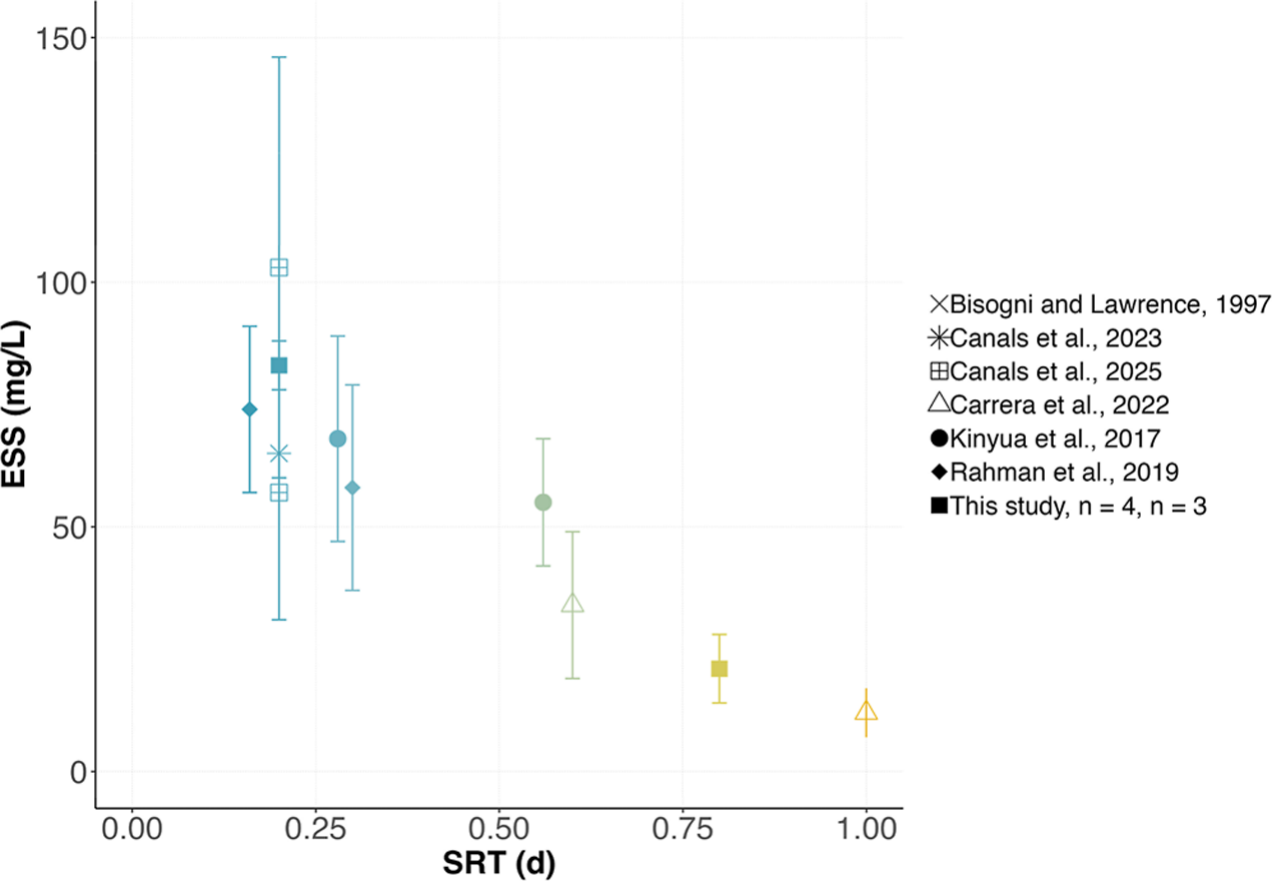
Change in the ESS concentration as a function of the SRT. Data presented are from this study and from the literature. Bars represent standard deviations when available, and “*n*” represents the number of measurements. Rahman et al.^19^ measured ESS in mgVSS/L and Kinyua et al.^17^ measured ESS during SVI_30_ tests. Raw wastewater was used as influent in all studies except in ours for which primary effluent wastewater was used.

#### 3.2.3 Sludge Volume Index at 30 min (SVI30)

SVI_30_ was measured at 0.2 and 0.8 d SRT and compared to literature results, to evaluate whether SRT and thus the flocs morphology have an impact on sludge settleability/compressibility (Figure 5). In our study, SVI_30_ largely increased with an increase in SRT: from 85 ± 57 mL/gTSS (*n* = 4) to 412 ± 84 mL/gTSS (*n* = 3) as SRT increased from 0.2 to 0.8 d. Based on data from the literature, a similar trend is observed, with SVI_30_ increasing linearly from 75 to 300 mL/g for a SRT increase from 0.16 and 1.0 d. The SVI–SRT trend derived from literature data suggests an SVI_30_ value of approximately 200 mL g^–1^ TSS at 0.8 days of SRT. Therefore, the SVI30 value monitored in our study significantly exceeds this expected value. The low compaction of the sludge grown at 0.8 d SRT is, however, consistent with the observation of large and heterogeneous flocs (Figure 3).

**Figure 5:**
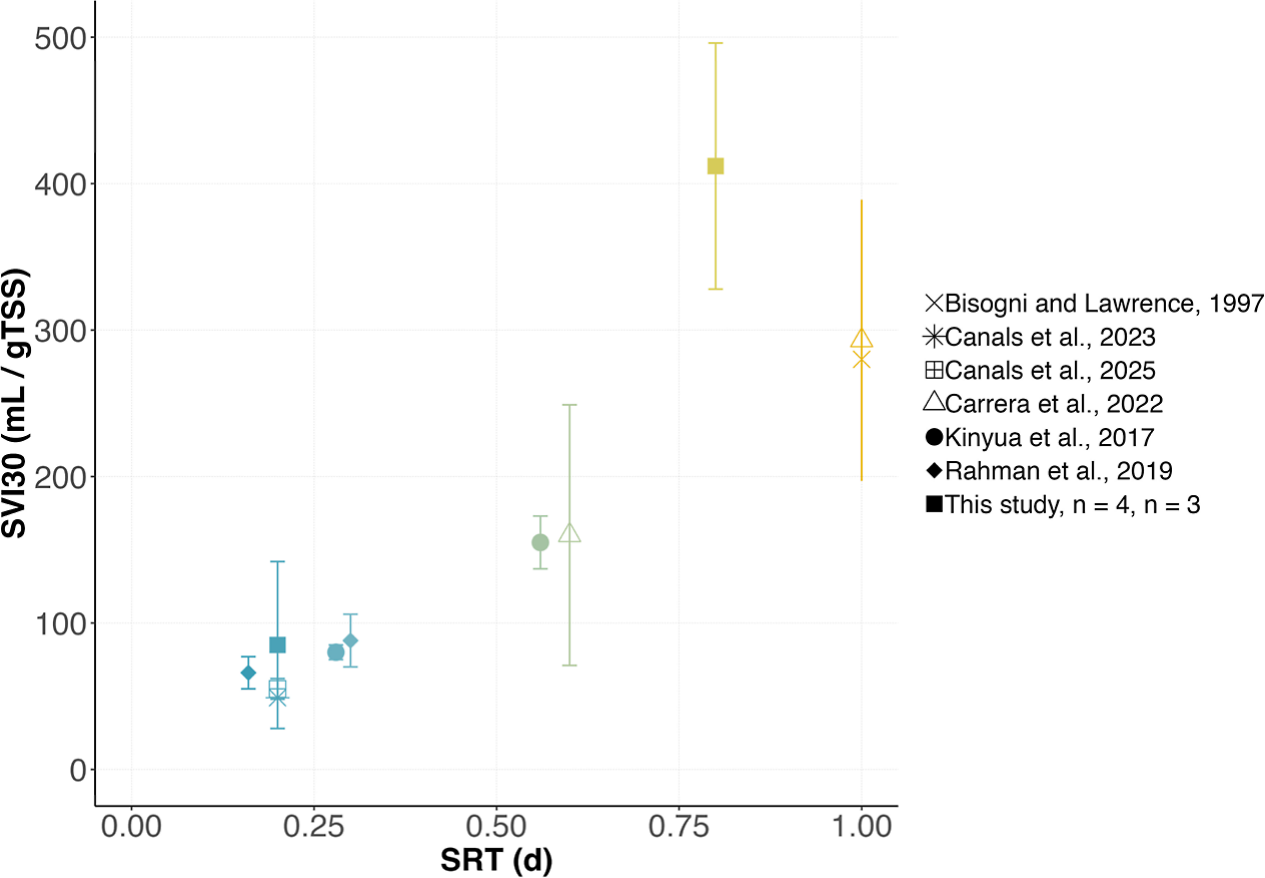
Change in SVI_30_ as a function of SRT. Data presented are from this study and the literature. Bars represent standard deviation when available and “*n*” the number of measurements. Raw wastewater was used as influent in all studies except in ours and studies from Mancell-Egala et al.^33^ and Van Winckel et al.^7^ for which primary effluent wastewater was used.

#### 3.2.4 Bioflocculation Capacity (TODF and TOF)

TODF and TOF curves were generated for sludge grown at 0.2 and 0.8 days in the HRAS system (Figure 6). A curve obtained for a conventional activated sludge (CAS) grown at 15 d SRT is provided as a reference of a sludge with good bioflocculation properties (Figure 6). For the CAS, the supernatant TSS slightly decreased with an increase in initial TSS concentration up to 200 mg/L, after which a distinct change in the slope was observed, with a plateau in the supernatant TSS as the initial TSS concentration increased further. TODF and TOF values of 154 and 429 mgTSS/L, respectively, were quantified for the CAS. In contrast, the TOF curves of HRAS grown at 0.2 and 0.8 d SRT exhibited a very different trend compared to the CAS curve and did not present any clear slope break indicative of a pronounced bioflocculation. As the initial TSS concentration increased, the supernatant TSS gradually deviated from it, while such deviation was already observed at a low TSS concentration (<50 mgTSS/L). Unlike for the CAS, only TODF values were quantified for the HRAS. The sludge grown at 0.2 and 0.8 d SRT was characterized by average TOFD values of 40 ± 17 (*n* = 4) and 16 ± 9 mgTSS/L (*n* = 3), respectively (*p*-value = 0.052).

**Figure 6:**
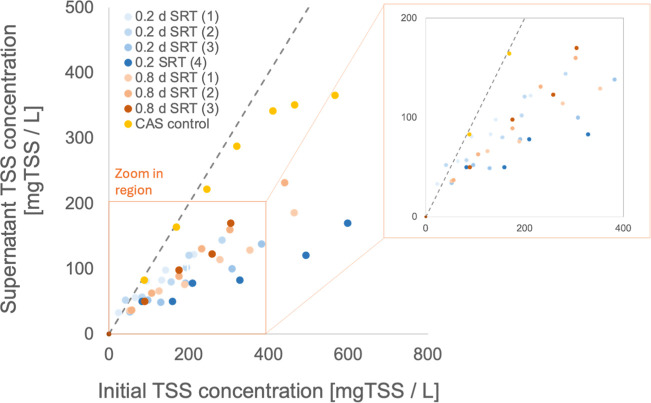
Change in the supernatant TSS as a function of initial TSS concentration (TODF and TOF curves) of HRAS pilot and a CAS process.

### 3.3 How Does SRT Influence the Content and Composition of Sludge from HRAS Systems?

Our previous results demonstrate that a low SRT (0.2 days) limits bioflocculation, resulting in partial organic matter harvesting. A main question is therefore whether differences in the LB- and TB-polymeric substances of the sludge grown at 0.2 and 0.8 days of SRT could explain the differences in sludge bioflocculation and settleability that we observed. The concentration and composition of the different LB- and TB-extracts were analyzed and compared to literature results (Figure 7).

**Figure 7:**
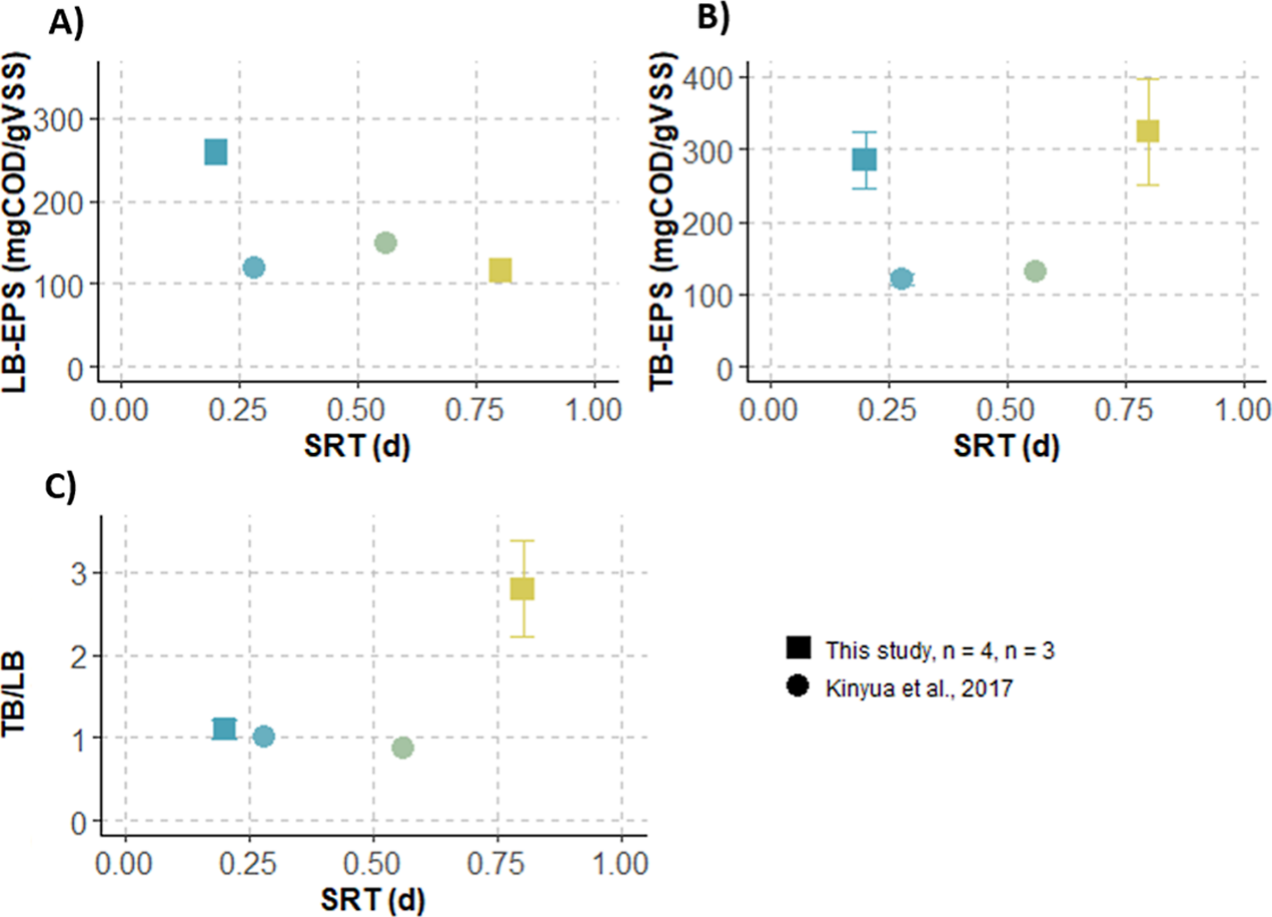
Change in LB- and TB-polymeric substances concentration as a function of SRT. Data presented are from this study and the literature. Bars represent standard deviation and “*n*” the number of measurements. Standard deviation was measured for each point but was often too low to be visible on the graph.

A clear effect of the SRT on the solubilization of LB- and TB-polymeric substances from the sludge was observed (Figure 7A,B). In our study, a very clear decrease in the LB-polymeric substances content was observed with an increase in SRT: from 259 ± 15 (*n* = 4) at 0.2 d SRT to 116 ± 9 mgCOD/gVSS (*n* = 3) at 0.8 d SRT (Welch’s *t*-test, *p*-value = 3 × 10^–5^). A linear regression performed on the mean LB-PS content from both our study and the literature suggested a decreasing trend with increasing SRT, although this relationship was only slightly significant (*p* = 0.059), potentially due to the small sample size (*n* = 4 mean values). In contrast, our measurements indicate that TB-polymeric substances content remained on the other hand rather stable with values of 285 ± 38 (*n* = 4) to 324 ± 73 mgCOD/gVSS (*n* = 3) measured at 0.2 and 0.8 d SRT, respectively (Welch’s *t*-test, *p*-value = 0.46). A linear regression on the mean values from our study and the literature confirmed the absence of a trend between the TB-PS content and the SRT. The decrease in LB-PS content, combined with the stability of TB-PS content, led to an increase in the TB/LB ratio from 1.1 ± 0.12 (*n* = 4) to 2.8 ± 0.6 (*n* = 3) (Figure 7C). A linear regression on all data indicated a statistically significant increase in the TB/LB ratio with an increasing SRT (*p*-value = *p*-value = 0.03). A linear regression performed on the mean ratio confirmed a strong correlation between the TB/LB and SRT (*p*-value = 0.017).

Additionally, our estimates of the slowly biodegradable substrate content in the sludge showed significant variations depending on the SRT. X_S_ represented the dominant fraction of the sludge grown at 0.2 d SRT sludge, with a fraction varying from 0.92 to 0.63 when the *k*_hyd_ value was increased from 0.5 to 3 d^–1^. For a SRT of 0.8 d, X_S_ represents a lower fraction of the sludge, with a fraction decreasing from 0.86 to 0.36 as the *k*_hyd_ value increases from 0.5 to 3 d^–1^.

The biochemical composition of the LB- and TB-extracts was further investigated with regard to the molecular weight distribution. Biopolymers contents (MW > 20 kDa), expressed in percentage of the organic carbon content of the LB- and TB-extracts, are shown in Figure 8. Overall, the LB- and TB-extracts of the sludge grown at 0.2 and 0.8 days SRT contained 20 to 35% of biopolymers (relative to organic carbon). The LB- and TB-extracts were therefore dominantly composed of molecules with a molecular weight lower than 20 kDa and down to 500 Da. At 0.2 d SRT, LB- and TB-extracts contained approximately the same percentage of biopolymers, 25 ± 2% (*n* = 4) and 27 ± 6% (*n* = 4), respectively. At 0.8 d SRT, the biopolymer content of LB-extract was double of the one of the TB-extract, with values of 36 ± 6% (*n* = 3) and 18 ± 8% (*n* = 3), respectively. Additionally, the protein content of the extracts increased with longer SRTs, especially in the TB-extract. At an SRT of 0.2 days, the protein content was 67 ± 35 mgN/gC (*n* = 4) in the LB-extract and 44 ± 27 mgN/gC (*n* = 4) in the TB-extract. At a higher SRT of 0.8 days, the protein content increased to 86 ± 30 mgN/gC (*n* = 3) in the LB-extract and especially to 147 ± 21 mgN/gC (*n* = 3) in the TB-extract.

**Figure 8:**
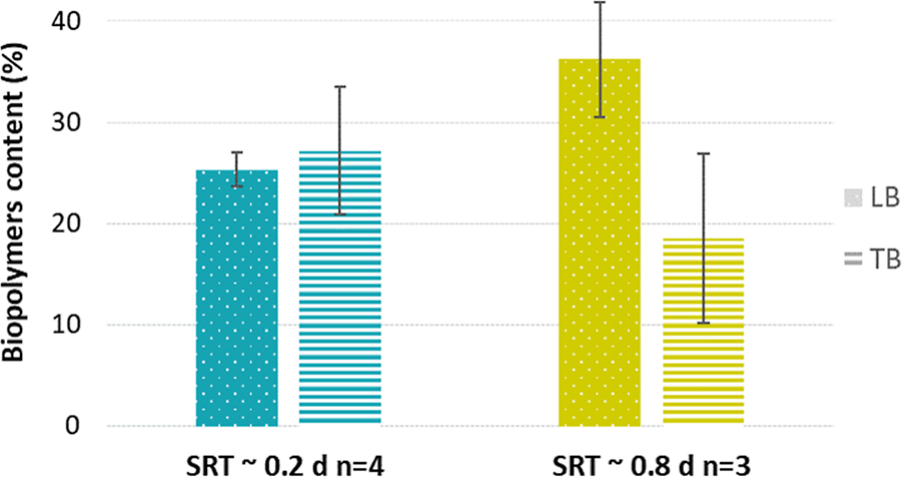
Biopolymers content (%) in LB- and TB-extracts at 0.2 and 0.8 d SRT (mgC-based). Bars represent standard deviation and “*n*” the number of measurements.

### 3.4 Bioflocculation Limits Organic Matter Harvesting in HRAS Systems

In our study, the bioflocculation mechanism in HRAS was investigated under contrasting SRT conditions using primary effluent wastewater to strengthen the relevance of our analysis. We applied contrasted SRT values (0.2 and 0.8 days) that fall within the relevant range typically applied when operating HRAS systems. A main result of our study is that organic matter harvesting by HRAS systems operated at low SRT is limited by poor bioflocculation, despite a high organic matter redirection into biomass. Our results indeed indicate that a 0.2 d SRT helps minimizing the organic matter oxidation, thus increasing its redirection into biomass (Figure 2). Such a link between the SRT and the oxidation process/biomass production is well-understood and has been already reported in the literature. However, previous studies on HRAS were performed on a narrow range of SRT values, resulting in almost similar system performances.^17,19^ For instance, Kinyua et al.^17^ and Rahman et al.^19^ focused on a narrower range of SRT, limiting their ability to observe contrasting system responses (0.28–0.56 days and 0.16–0.3 d, respectively). In contrast, our approach complements the studies of Carrera et al.^30^ and Jimenez et al.,^16^ who also examined a broader SRT range. These studies do not allow for a clear relationship of the bioflocculation and the systems performances (redirection and harvesting efficiencies). On the contrary, our results were obtained under two contrasting SRT conditions (0.2 and 0.8 days), representing the extremes of the conventional SRT range of HRAS systems, while using primary effluent WW to specifically study the bioflocculation. Our findings demonstrate that increasing the redirection of organics toward biomass production does not lead to a proportional increase in organic matter harvesting. In other words, while maintaining a low SRT is effective in increasing the production of total biomass (active biomass and particulate substrate), this biomass is only partially harvested, as a significant fraction of it is lost via the effluent. As a consequence, the harvesting efficiency of COD_harv_ measured in our study increased only slightly from 35 ± 6 to 42 ± 4% when SRT was reduced from 0.8 to 0.2 d, despite redirection increasing by a factor of almost 3 (Figure 2). When operated at 0.2 day SRT, a significant loss of organic matter actually occurred via the HRAS effluent. Such partial harvesting efficiency was also reported in the literature for HRAS systems operated at low SRT.^16,17,19,30^ In our study, such loss of organic matter via the effluent was observed despite using a secondary clarifier with a low SOR (0.7 m^3^/m^2^/d). A low SOR can, in principle, help balance the poor settleability of HRAS, thus increasing harvesting.^32^ A main point to discuss is therefore what mechanism is responsible for the limited increase in harvesting, as compared to the pronounced increase in the redirection.

Both poor bioflocculation and/or poor settling properties of a sludge can result in a significant loss of organic matter via the effluent. Bioflocculation is defined as the floc formation, allowing particles to settle.^5^ A poor bioflocculation implies that small particles and colloids are not fully captured, resulting in high ESS concentrations.^6^ A poor settleability of the sludge can also result in significant TSS overflow through the secondary clarifier. Several observations made in our study consistently support that the bioflocculation of the sludge grown at a low 0.2 d SRT was limited, e.g., high ESS concentrations (Figure 4), high COD_eff_ fraction (Figure 2), low pCOD and cCOD removals (Figure 1), and the absence of a clear threshold of flocculation. In HRAS systems fed with primary clarifier effluent, like in our study, the influent particulate COD consists only of small diameter particles that are slowly settling and therefore passed through the primary clarifier, while most of the colloids are removed through its capture by the sludge.^5^ The settling velocity of these particles/colloids is therefore too small to allow them to settle individually, and their retention in the HRAS system thus relies on bioflocculation. In our study, we consistently measured elevated pCOD concentrations in the effluent of the system operated at 0.2 day SRT, therefore indicative of a poor bioflocculation capacity of the sludge grown under those conditions. The presence of fluffier and more developed flocs observed at 0.8 d SRT (Figure 3) also suggests that bioflocculation is improved at higher SRT. In the case of aerobic granules, a significant adsorption of particles onto the granule surface leads to the formation of hairy/fluffy granules due to important growth of fast growing heterotrophs on hydrolysis products.^34^ It can be suggested that the hairy structure of flocs grown at 0.8 d of SRT is indicative of a high adsorption of particles, i.e., a high bioflocculation capacity. In turn, fluffy floc structures could promote further bioflocculation, by providing numerous binding sites and by increasing collision frequency due to the large surface area. Bioflocculation of sludge grown at low SRT might have additionally been limited by the low MLSS which reduces the collision rate,^8^ while the collision efficiency might have been impacted by the specific EPS fingerprint of this sludge (see the following section). Finally, the poorer bioflocculation capacities at low SRT are also suggested by our measurements of TODF. Introduced by Mancell-Egala et al.^33^ and further developed by Fau et al.,^28^ this method is applied in our study for the first time to distinguish and quantify the minimal TSS concentration at which sedimentation “begins” (TODF) or “accelerates” due to bioflocculation (TOF). In our study, the TODF values of sludge grown at an SRT of 0.2 days were statistically higher than those of sludge grown at an SRT of 0.8 days. This difference can be explained by the presence of larger and denser flocs in the 0.8 day sludge (Figure 3). According to sedimentation theory, particles with higher density and greater size exhibit higher settling velocities (as described by Stokes’ law for laminar conditions). Thus, at an equivalent initial TSS concentration, a sludge containing a fraction of such fast-settling flocs would yield a lower residual supernatant TSS concentration after settling, resulting in a lower TODF. Additionally, no clear slope break was observed in the TODF and TOF curves for either sludge; i.e., TOF was not quantifiable, indicating that pronounced bioflocculation leading to accelerated sedimentation did not occur (Figure 6). Fau et al.^28^ already suggested that the protocol of TODF and TOF quantification that is based on a critical settling velocity of 1.5 m/h (i.e., a value 15% lower than the SOR of 1.7 m^3^/m^2^/h at which failure of the secondary clarifier of the CAS system is often reported^33^) is not well-adapted to the HRAS system. For the HRAS system, the failure of the secondary clarifier often occurs at SOR ≤ 1 m^3^/m^2^/h.^5,32^ In our study, the absence of observation of a slope break on the TOF curves further supports the need to adopt a lower critical settling velocity during these tests—more representative of HRAS performance—to accurately capture their bioflocculation capacity (e.g., 0.85 m^3^/m^2^/h, i.e., 15% below the SOR of 1 m^3^/m^2^/h reported by Van Winckel et al.^5^) in addition to covering a larger range of initial TSS concentration. Ultimately, while previous literature has suggested that bioflocculation is limited at very low SRT, our study provides metrics and clear evidence supporting a fundamental difference in the bioflocculation capacity of HRAS (absence of TOF, low retention of particulate, and colloidal COD during treatment of primary effluent WW).

A poor settleability of the sludge can also limit harvesting, in addition to the effect of poor bioflocculation. However, SVI_30_ values significantly lower at 0.2 than at 0.8 d SRT indicate a better compressibility of the sludge grown under low SRT conditions (Figure 5). In our study, the HRAS system was fed with the primary effluent wastewater. The low SVI_30_ at 0.2 d SRT is therefore not explained by an increased ballasted settling, due to the accumulation of fast-settling influent solids in the sludge. On the contrary, the high SVI_30_ at 0.8 d SRT likely resulted from the presence of large and fluffy flocs preventing a good compressibility (Figure 3). Such a high SVI value did not yield elevated ESS concentrations, which might be explained by the rather large surface area of the secondary clarifier (and therefore the low SOR: 0.7 m^3^/m^2^/d). One may ultimately keep in mind that a good compressibility is different from a high settling velocity.

All our results thus lead to the conclusion that a low SRT promotes organic matter redirection but prevents a good bioflocculation/sedimentation, thus limiting the organic matter harvesting and its further conversion into energy/valuable products. EPS are a key component of bacterial aggregates and are associated with various functions: the (ad)­sorption of organic matter, the formation of their 3-dimensional architecture of the flocs, etc.^35^ It has been proposed that the quantity, composition, and functions of the EPS of sludge grown in HRAS systems govern the bioflocculation and therefore harvesting efficiency.^17^ A main question is therefore to understand how the EPS content and composition influence the bioflocculation mechanisms.

### 3.5 Enrichment of Loosely Bound Polymeric Substances at Low SRT Correlates with Poor Bioflocculation

A second key finding of our study is that a low SRT (0.2 d) promotes the growth of a sludge enriched with LB polymeric substances characterized by a low biopolymer content (25–30%) (Figures 7 and 8), which correlates with poor bioflocculation capacity. A main aspect to discuss is therefore the relationship between growth conditions (in terms of SRT), solubilized polymeric substances, and their impact on bioflocculation.

Based on the selected extraction method of our study, polymeric substances that are weakly attached to aggregates and readily solubilized are classified as “loosely bound” (LB). All polymeric substances, whether of microbial origin (EPS) or derived from the slowly biodegradable substrate accumulated in the sludge, can in principle be (partially) solubilized by the extraction method and subsequently recovered in the LB extracts. In our study, estimation of X_S_ content indicated that the sludge grown at an SRT of 0.2 days was predominantly composed of a slowly biodegradable substrate from the influent (63% COD-based at a *k*_hyd_ value of 3 d^–1^), while its LB polymeric substances content was also high (>250 mg COD/L). Increasing the SRT to 0.8 days led to a substantial reduction in both the slowly biodegradable substrate content (35%) and in the LB-polymeric substances (116 ± 9 mgCOD/gVSS) content of the sludge. A large proportion of X_S_ in the sludge thus correlates with a large content of LB polymeric substances. We therefore suggest that the high LB content of the sludge grown at 0.2 d SRT originates from both the EPS matrix of the flocs (microbially produced) and, more importantly, from the solubilization of the influent slowly biodegradable substrate which accumulates extensively in the sludge under these operating conditions. One may also expect that the flocculation properties of solubilized EPS differ from those of EPS derived from sludge with a lower X_S_ content. Collision efficiency, a key mechanism influencing sludge bioflocculation,^9^ is affected by sludge properties such as charge, size, and hydrophobicity. It is therefore plausible that the specific EPS fingerprint of sludge grown at 0.2 d of SRT influenced this mechanism and the resulting bioflocculation. Ultimately, both the solubilization of EPS from X_S_ and the distinct flocculation capacity of EPS derived from X_S_-rich sludge require further investigation to clearly demonstrate their impact on bioflocculation and HRAS performance.

Consequently, the sludge grown at 0.2 d SRT consisted mostly of poorly developed flocs that were significantly smaller than those formed at 0.8 d SRT and that were characterized by a lower LB polymeric substance content (Figure 3). Similar observations have been reported by Feng et al.^36^ and Liao et al.,^37^ who reported on the link between the LB polymeric substance content and the bioflocculation capacity of conventional activated sludge. These authors found a negative correlation between LB polymeric substances and aggregate size, possibly because these specific polymeric substances weaken cell attachment.^23,36^ We argue that the composition of LB polymeric substances of the 0.2 d SRT sludge was dominantly composed of polymeric substances derived from the solubilization of the slowly biodegradable substrate and that these polymeric substances were associated with poor bioflocculation capability, as these polymeric substances are likely not structural EPS produced by the bacteria to form flocs. Increasing the SRT from 0.2 to 0.8 d helps increasing the time for the heterotrophic biomass to grow (supported by the increasing oxidation of COD and also by the increasing protein content of the extracts), while also providing more time for the hydrolysis of Xs (therefore not solubilized by the EPS extraction method). Biopolymers such as proteins and polysaccharides hold flocculant properties thanks to their long molecular chains.^13,38^ Our SEC-OCD-OND results showed that the biopolymer content of the LB extracts increased with increasing SRT (Figure 8), which could potentially explain the better floc formation and the slightly improved bioflocculation observed with the sludge grown at 0.8 days of SRT, although overall bioflocculation remained limited. No differences between the biopolymer contents of TB-extracts were observed (Figure 8). However, the protein content of these extracts significantly increased with an SRT increase (from 44 ± 27 to 147 ± 21 mgN/gC), supporting the understanding that TB polymeric substances are strongly associated with bacteria and that their accumulation is linked to increased bacterial growth at higher SRTs.

Overall, a key contribution of this work was to demonstrate that a low SRT (0.2 days) promotes the development of sludge enriched in LB-polymeric substances, likely primarily associated with a slowly biodegradable substrate originating from the influent. These nonmicrobially synthesized polymeric substances exhibit poor bioflocculation capacity, ultimately limiting the efficient harvesting of organic matter. But our results also highlight the difficulty in linking general indicators, such as LB-/TB-polymeric substance content and biopolymer content, to the bioflocculation capacity of sludge, as also suggested in recent studies.^14,21,36,39^ Instead, we emphasize the need for a more detailed characterization of the composition and flocculating functions of EPS to gain a deeper understanding of their roles in the bioflocculation mechanism.

### 3.6 Practical Implications

Our study demonstrates that organic matter harvesting by HRAS systems operated at very low SRT (e.g., 0.2 d) is hampered by bioflocculation, ultimately resulting in a similar harvesting efficiency to the one of systems operated at a higher SRT (e.g., 0.8 d). Operating HRAS systems at low SRT is usually recommended to decrease the energy demand. But lowering the SRT of HRAS systems also results in a loss of organic matter via the effluent, thus reducing the potential energy production. If harvesting efficiency were almost total, COD_was_ would increase to around 70%, which would correspond to a very high recovery of influent COD. Increasing the capture of influent COD by HRAS systems thus requires having a better control on the bioflocculation or to not rely on such a mechanism. Canals et al.^32^ reported that reducing the surface overflow rate of the clarifier from 1.6 to 0.8 m h^–1^ helps increasing the influent SS removal from 71 to 85%. However, increasing clarifier design to reduce the surface overflow rate is not a practical solution as it would require a significantly larger footprint, which is often infeasible in the existing WWTP infrastructure. A second option would be to provide conditions for the bacteria to produce EPS with good flocculant properties. Adapting process configuration in order to create specific growth conditions, such as in a contact-stabilization system, has been suggested as a relevant approach to control EPS production.^19^ However, this requires a much more detailed understanding on how to drive the microbial response toward the secretion of EPS with desired functions. A second option is to use chemicals with flocculant properties.^5^ Although petroleum-based flocculants have proved effective, an assessment of the impact of chemicals on sludge recovery and economic/environmental viability would be necessary.^40^ A relevant route to explore is potentially the use of biosourced flocculant agents.^38^ Finally, a last option is to capture organic matter through microsieving, as a replacement of secondary clarification, which is a relevant option because screens represent an ideal physical barrier for solids removal and can be operated with a mesh-size as small as 15 μm. A microsieve is a relevant alternative to primary clarifiers, as they offer various advantages: (1) microsieves rely on physical retention rather than retention via sedimentation, (2) they can be combined with flocculants, if needed, to increase harvesting,^41^ and (3) they are compact and low-maintenance and are mechanically very robust. We advocate that further research efforts should be dedicated to evaluating microsieves as a viable alternative to secondary clarifiers for biomass harvesting in HRAS systems, particularly under conditions where settleability is limited.

## 4 Conclusions

- Reducing SRT of HRAS systems promotes the accumulation of a slowly biodegradable substrate (X_S_) in the sludge, leading to a distinct fingerprint of loosely and tightly bound polymeric substances (LB- and TB-PS). This shift in LB-/TB-PS content correlates with a limited bioflocculation and specific floc morphology impacts settling and ultimately hampers biomass harvesting.
- Reducing the SRT from 0.8 to 0.2 d SRT results in an increase in the X_S_ content of the sludge, from 35 to >60% (based on COD mass balance). Such increase in Xs content correlates with a higher content in LB-polymeric substances (from 116 to 260 mgCOD/gVSS, respectively) and a low TB/LB ratio (from 2.8 at 0.8 d to 1.1. at 0.2 d). In turn, the sludge grown at 0.2 d SRT was mostly composed of small flocs and associated with a poor bioflocculation capability, resulting in a significant loss of ESS in the effluent.
- Low SRT enhances organic matter redirection but impairs bioflocculation therefore limiting harvesting, as a result of the specific EPS fingerprint and floc morphology. Operating HRAS at a low SRT (0.2 days) effectively minimizes organic matter oxidation, thereby increasing organic matter redirection into biomass. The increased redirection into biomass, however, did not result in a proportional increase in harvesting due to the poor sludge bioflocculation. Similar harvesting efficiencies of around 40% were thus observed for the HRAS systems operated at 0.2 or 0.8 SRT.
- Maximizing the harvesting of organic matter with HRAS systems requires better bioflocculation control or the use of alternative capture methods, e.g., by increasing the secretion of EPS with good flocculant properties at low SRT or by combining HRAS with chemical flocculants or microsieving.

## Supplementary Material



## Notes

Additional stereomicroscopic pictures of flocs and detailed mass balance on slowly biodegradable substrate X_S_ and associated assumptions (PDF)

## References

[1] Modin O., Persson F., Wilen B. M., Hermansson M. (2016). Nonoxidative
removal of organics in the activated sludge process. Crit. Rev. Environ. Sci. Technol..

[2] Guven H., Dereli R. K., Ozgun H., Ersahin M. E., Ozturk I. (2019). Towards sustainable
and energy efficient municipal wastewater treatment by up-concentration
of organics. Prog. Energy Combust..

[3] Guthi R. S., Tondera K., Gillot S., Buffière P., Boillot M., Chazarenc F. (2022). A-Stage process-Challenges and drawbacks
from lab to full scale studies: A review. Water
Res..

[4] Sancho I., Lopez-Palau S., Arespacochaga N., Cortina J. L. (2019). New concepts on
carbon redirection in wastewater treatment plants: A review. Sci. Total Environ..

[5] Van
Winckel T., Ngo N., Sturm B., Al-Omari A., Wett B., Bott C., Vlaeminck S. E., De Clippeleir H. (2022). Enhancing bioflocculation in high-rate activated sludge
improves effluent quality yet increases sensitivity to surface overflow
rate. Chemosphere.

[6] Rahman A., Meerburg F. A., Ravadagundhi S., Wett B., Jimenez J., Bott C., Al-Omari A., Riffat R., Murthy S., De Clippeleir H. (2016). Bioflocculation management through high-rate contact-stabilization:
A promising technology to recover organic carbon from low-strength
wastewater. Water Res..

[7] Van
Winckel T., Liu X. C., Vlaeminck S. E., Takács I., Al-Omari A., Sturm B., Kjellerup B. V., Murthy S. N., De Clippeleir H. (2019). Overcoming floc formation limitations
in high-rate activated sludge systems. Chemosphere.

[8] AlSayed A., Soliman M., ElDyasti A. (2023). Mechanistic
assessment reveals the
significance of HRT and MLSS concentration in balancing carbon diversion
and removal in the A-stage process. J. Environ.
Manage..

[9] Alsayed A., Soliman M., Eldyasti A. (2023). The A-stage
process to promote bioflocculation
and microbial storage for carbon redirection: current perspectives
and future research directions. Rev. Environ.
Sci. Bio..

[10] Suresh A., Grygolowicz-Pawlak E., Pathak S., Poh L. S., Majid M. B. A., Dominiak D., Bugge T. V., Gao X., Ng W. J. (2018). Understanding
and optimization of the flocculation process in biological wastewater
treatment processes: A review. Chemosphere.

[11] Christensen B. E. (1989). The Role of Extracellular
Polysaccharides
in Biofilms. J. Biotechnol..

[12] Nouha K., Kumar R. S., Balasubramanian S., Tyagi R. D. (2018). Critical review of EPS production, synthesis and composition
for sludge flocculation. J. Environ. Sci..

[13] Sheng G. P., Yu H. Q., Li X. Y. (2010). Extracellular
polymeric substances
(EPS) of microbial aggregates in biological wastewater treatment systems:
a review. Biotechnol. Adv..

[14] Liao B. Q., Allen D. G., Droppo I. G., Leppard G. G., Liss S. N. (2001). Surface
properties of sludge and their role in bioflocculation and settleability. Water Res..

[15] Urbain V., Block J. C., Manem J. (1993). Bioflocculation in
activated sludge:
an analytic approach. Water Res..

[16] Jimenez J., Miller M., Bott C., Murthy S., De Clippeleir H., Wett B. (2015). High-rate activated
sludge system for carbon management - Evaluation
of crucial process mechanisms and design parameters. Water Res..

[17] Kinyua M. N., Elliott M., Wett B., Murthy S., Chandran K., Bott C. B. (2017). The role of extracellular
polymeric substances on carbon
capture in a high rate activated sludge A-stage system. Chem. Eng. J..

[18] Meerburg F. A., Boon N., Van Winckel T., Pauwels K. T., Vlaeminck S. E. (2016). Live Fast,
Die Young: Optimizing Retention Times in High-Rate Contact Stabilization
for Maximal Recovery of Organics from Wastewater. Environ. Sci. Technol..

[19] Rahman A., De Clippeleir H., Thomas W., Jimenez J. A., Wett B., Al-Omari A., Murthy S., Riffat R., Bott C. (2019). A-stage and
high-rate contact-stabilization performance comparison for carbon
and nutrient redirection from high-strength municipal wastewater. Chem. Eng. J..

[20] Liao B. Q., Lin H. J., Langevin S. P., Gao W. J., Leppard G. G. (2011). Effects
of temperature and dissolved oxygen on sludge properties and their
role in bioflocculation and settling. Water
Res..

[21] Chen X. Q., Kong F. G., Fu Y. J., Si C. L., Fatehi P. (2019). Improvements
on activated sludge settling and flocculation using biomass-based
fly ash as activator. Sci. Rep..

[22] Li X. Y., Yang S. F. (2007). Influence of loosely bound extracellular
polymeric
substances (EPS) on the flocculation, sedimentation and dewaterability
of activated sludge. Water Res..

[23] Liu Y., Fang H. H. P. (2003). Influences of
extracellular polymeric substances (EPS)
on flocculation, settling, and dewatering of activated sludge. Crit. Rev. Environ. Sci. Technol..

[24] Meerburg F. A., Vlaeminck S. E., Roume H., Seuntjens D., Pieper D. H., Jauregui R., Vilchez-Vargas R., Boon N. (2016). High-rate activated sludge communities
have a distinctly different
structure compared to low-rate sludge communities, and are less sensitive
towards environmental and operational variables. Water Res..

[25] Liu X. M., Sheng G. P., Luo H. W., Zhang F., Yuan S. J., Xu J., Zeng R. J., Wu J. G., Yu H. Q. (2010). Contribution of
Extracellular Polymeric Substances (EPS) to the Sludge Aggregation. Environ. Sci. Technol..

[26] APHA . Standard Methods for the Examination of Water and Wastewater. 20th ed.; American Public Health Association: Washington, DC, 1998.

[27] Mamais D., Jenkins D., Prrr P. (1993). A rapid physical-chemical
method
for the determination of readily biodegradable soluble COD in municipal
wastewater. Water Res..

[28] Fau Z., Damien T., Azais A., Derlon N., Chazarenc F., Gillot S. (2024). Towards a standardised
analysis of experimental Threshold
Of Flocculation (TOF) curves. Water Sci. Technol..

[29] Lim K., Parameswaran P. (2022). Critical evaluation
of heat extraction temperature
on soluble microbial products (SMP) and extracellular polymeric substances
(EPS) quantification in wastewater processes. Water Sci. Technol..

[30] Carrera J., Carbó O., Doñate S., Suárez-Ojeda M. E., Pérez J. (2022). Increasing the energy production in an urban wastewater
treatment plant using a high-rate activated sludge: Pilot plant demonstration
and energy balance. J. Clean. Prod..

[31] Canals J., Cabrera-Codony A., Carbó O., Torán J., Martín M., Baldi M., Gutiérrez B., Poch M., Ordóñez A., Monclús H. (2023). High-rate
activated sludge at very short SRT: Key factors for process stability
and performance of COD fractions removal. Water
Res..

[32] Canals J., Cabrera-Codony A., Carbó O., Turolla A., García S., Lema J. M., Monclús H. (2025). Fostering the performance and stability
of the high-rate activated sludge process: Biomass dynamics, oxygen
consumption and clarifier operation. J. Environ.
Chem. Eng..

[33] Mancell-Egala W., De Clippeleir H., Su C., Takacs I., Novak J. T., Murthy S. N. (2017). Novel Stokesian
Metrics that Quantify Collision Efficiency,
Floc Strength, and Discrete Settling Behavior. Water Environ. Res..

[34] de Kreuk M.
K., Kishida N., Tsuneda S., van Loosdrecht M. C.
M. (2010). Behavior of polymeric
substrates in an aerobic granular
sludge system. Water Res..

[35] Flemming H. C., Wingender J. (2010). The biofilm matrix. Nat. Rev.
Microbiol..

[36] Feng Q., Tai X. R., Sun Y. Q., Li M. (2019). Influence of turbulent
mixing on the composition of extracellular polymeric substances (EPS)
and aggregate size of aerated activated sludge. Chem. Eng. J..

[37] Liao B. Q., Droppo I. G., Leppard G. G., Liss S. N. (2006). Effect of solids
retention time on structure and characteristics of sludge flocs in
sequencing batch reactors. Water Res..

[38] Salehizadeh H., Yan N. (2014). Recent advances in
extracellular biopolymer flocculants. Biotechnol.
Adv..

[39] Ngo K. N., Van Winckel T., Massoudieh A., Wett B., Al-Omari A., Murthy S., Takacs I., De Clippeleir H. (2021). Towards more
predictive clarification models via experimental determination of
flocculent settling coefficient value. Water
Res..

[40] Taboada-Santos A., Rivadulla E., Paredes L., Carballa M., Romalde J., Lema J. M. (2020). Comprehensive
comparison of chemically enhanced primary
treatment and high-rate activated sludge in novel wastewater treatment
plant configurations. Water Res..

[41] Väänänen J., Cimbritz M., la Cour
Jansen J. (2016). Microsieving in primary treatment:
effect of chemical dosing. Water Sci. Technol..

