# Supplementary material for: Impact of Slowly Biodegradable COD and Loosely Bound Polymeric Substances Accumulation in High-Rate Activated Sludge: Implications for Bioflocculation and Organic Matter Harvesting
Source: ACS ES T Eng. 2025 Oct 1;5(12):3632–44. doi: 10.1021/acsestengg.5c00745 (PMC12707229; doi:10.1021/acsestengg.5c00745)
Supplement: Supplementary file 1 [file ee5c00745_si_001.pdf]

SUPPLEMENTARY INFORMATION: Impact of slowly biodegradable COD and Loosely-Bound Polymeric Substances accumulation in High-Rate Activated Sludge: implications for bioflocculation and organic matter harvesting

Zoé Fau[a], Antonin Azais[a], Sylvie Gillot[a*], Florent Chazarenc[a] and Nicolas Derlon[b,*]

[a] INRAE, UR REVERSAAL, 5 rue de la Doua, 69625, Villeurbanne, France

[b] Eawag, Swiss Federal Institute of Aquatic Science and Technology, Ueberlandstrasse 133, 8600, Dübendorf, Switzerland

Emails of the authors:

zoe.fau@laposte.net

antonin.azais@inrae.fr

sylvie.gillot@inrae.fr

florent.chazarenc@inrae.fr

Email of the corresponding author: nicolas.derlon@eawag.ch

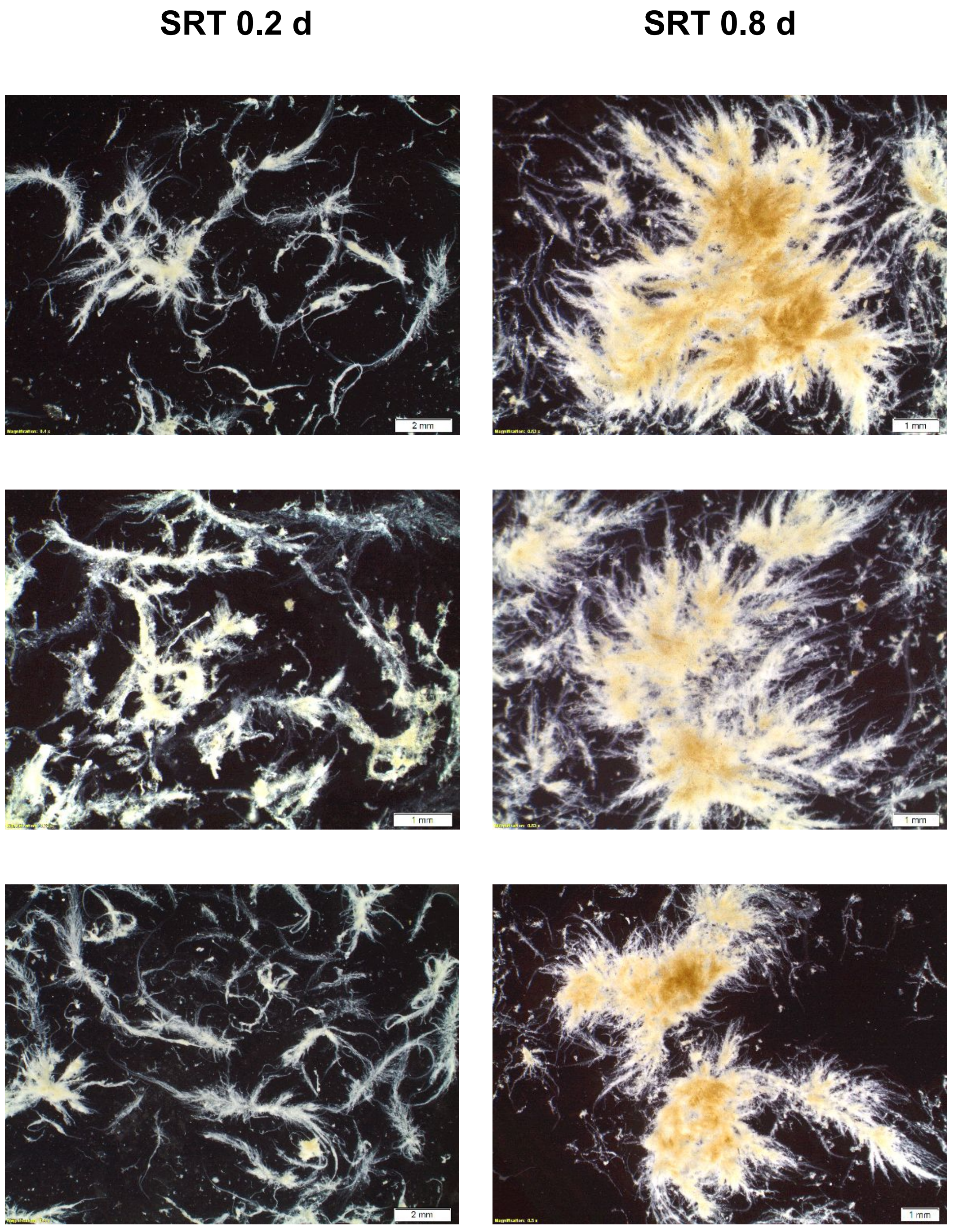

Figure SI.1: Stereomicroscopic images of flocs grown at 0.2 and 0.8 d SRT

Mass balance on Xs (concentration of **slowly biodegradable substrate** in the reactor):

$$V \cdot \frac{dX_s}{dt} = Q_{in} \cdot X_{s,in} - k_{hyd} \cdot X_s \cdot V - Q_{out} \cdot X_{s,out} - \frac{X_s \cdot V}{\theta_X} \qquad \text{Eq.1}$$

With:

Q_{in} the influent flow rate (m^3/d)
Q_{out} the effluent flow rate (m^3/d)
V the reactor volume (m^3)
X_s the concentration of slowly biodegradable substrate in the reactor (gCOD/m^3)
$X_{s,in}$ and $X_{s,out}$ the concentration of slowly biodegradable substrate in the influent and effluent, respectively (gCOD/m^3)
k_{hyd} the hydrolysis rate constant (d^{-1})
θ_X the solid resisdence time (d)

Eq.1 neglect the production of X_S from the lysis of biomass. At steady-state, the variation of concentration of slowly biodegradable substrate within the reactor ($\frac{dX_s}{dt}$) is nil, resulting in the following simplication of the equation:

$$k_{hyd} \cdot X_s \cdot V + Q_{out} \cdot X_{s,out} + X_S \cdot \frac{V}{\theta_X} = Q_{in} \cdot X_{s,in} \qquad \text{Eq.2}$$

Additionally, the ratio of the slowly biodegradable substrate concentration in the effluent to that in the reactor is equivalent to the ratio of the TSS concentration in the effluent to that in the reactor.

$$X_{s,out} = X_s \cdot \frac{X_{TSS,out}}{X_{TSS,r}} \qquad \text{Eq.3}$$

With:

$X_{TSS,out}$ the TSS concentration in the effluent (gTSS/L)
$X_{TSS,r}$ the TSS concentration in the reactor (gTSS/L)

$$k_{hyd} \cdot X_s \cdot V + Q_{out} \cdot \frac{X_{TSS,out}}{X_{TSS,r}} \cdot X_s + X_S \cdot \frac{V}{\theta_X} = Q_{in} \cdot X_{s,in} \qquad \text{Eq.4}$$

Dividing the equation above by Q_{in} and replacing the term V/$Q_{inf}$by θ_H (the hydraulic residence time), the following equation is proposed:

$$k_{hyd} \cdot X_s \cdot \theta_H + \frac{Q_{out}}{Q_{in}} \cdot \frac{X_{TSS,out}}{X_{TSS,r}} \cdot X_s + X_S \cdot \frac{\theta_H}{\theta_X} = X_{s,in} \qquad \text{Eq.5}$$

With:

θ_H the hydraulic residence time (d).

Therefore:

$$X_S \cdot \frac{\theta_H}{\theta_X} \cdot \left[k_{hyd} \cdot \theta_X + \frac{Q_{out}}{Q_{in}} \cdot \frac{X_{TSS,out}}{X_{TSS,r}} \cdot \frac{\theta_X}{\theta_H} + 1\right] = X_{s,in} \qquad \text{Eq.6}$$

$$X_S = \frac{\theta_X}{\theta_H} \cdot \frac{1}{\left[k_{hyd} \cdot \theta_X + \frac{Q_{out}}{Q_{in}} \cdot \frac{X_{TSS,out}}{X_{TSS,r}} \cdot \frac{\theta_X}{\theta_H} + 1\right]} \cdot X_{s,in} \qquad \text{Eq.7}$$

Table 1: Typical constant and variable values used for the estimation of Xs concentration (mgCOD / L).

Symbol	Description	Unit	Value	Reference
θ_X	Solid Residence Time	d	0.2 or 0.8	This study
θ_H	Hydraulic Residence Time	d	0.08	This study
k_{hyd}	Hydrolysis rate constant	d^{-1}	3	Henze et al. (2000)
Q_{in}	Influent flow rate	$m^3\ d^{-1}$	6.67	This study
Q_{out}	Effluent flow rate	$m^3\ d^{-1}$	3.95 (SRT = 0.2d)<br>6.06 (SRT = 0.8d)	This study
$X_{TSS,r}$	TSS concentration in reactor	kgTSS m^{-3}	0.4 (SRT = 0.2d)<br>1.3 (SRT = 0.8d)	This study
$X_{TSS,out}$	TSS concentration in effluent	kgTSS m^{-3}	0.083 (SRT = 0.2d)<br>0.021 (SRT = 0.8d)	This study
$X_{s,in}$	Slowly Biodegradable Substrate concentration in influent	kgCOD m^3	0.252 (SRT = 0.2d)<br>0.207 SRT = 0.8d)	Sum of the particulate and colloidal COD measured in this study.

One may notice that the hydrolysis rate constant of 3 d^{-1} is typically provided for Conventional Activated Sludge system operating at 20°C and several days SRT. It is unlikely that such value applies for HRAS systems characterised by a low DO and a very short SRT < 1d. While the hydrolysis rate constant of HRAS is unkown, one may expect this value to be lower than 3 d^{-1}. A sensitivity analysis on the effect of the k_{hyd} value on the fraction of Xs in the sludge was perform to evaluate the robustness of our conclusions. The fraction of Xs was calculated relative to the sludge total COD experimentally measured. This sensitivity analysis indicates that the main conclusions remain consistant for a k_{hyd} values ranging from 0.5 to 3 d^{-1}: the 0.2 d SRT sludge is dominantly composed of Xs (fraction varying from 0.92 to 0.63 as k_{hyd} increases) whily Xs represents a lower fraction of the sludge grown at 0.8 d SRT (fraction varying in a larger range, from 0.86 to 0.36 as k_{hyd} increases from 0.5 to 3d^{-1}).

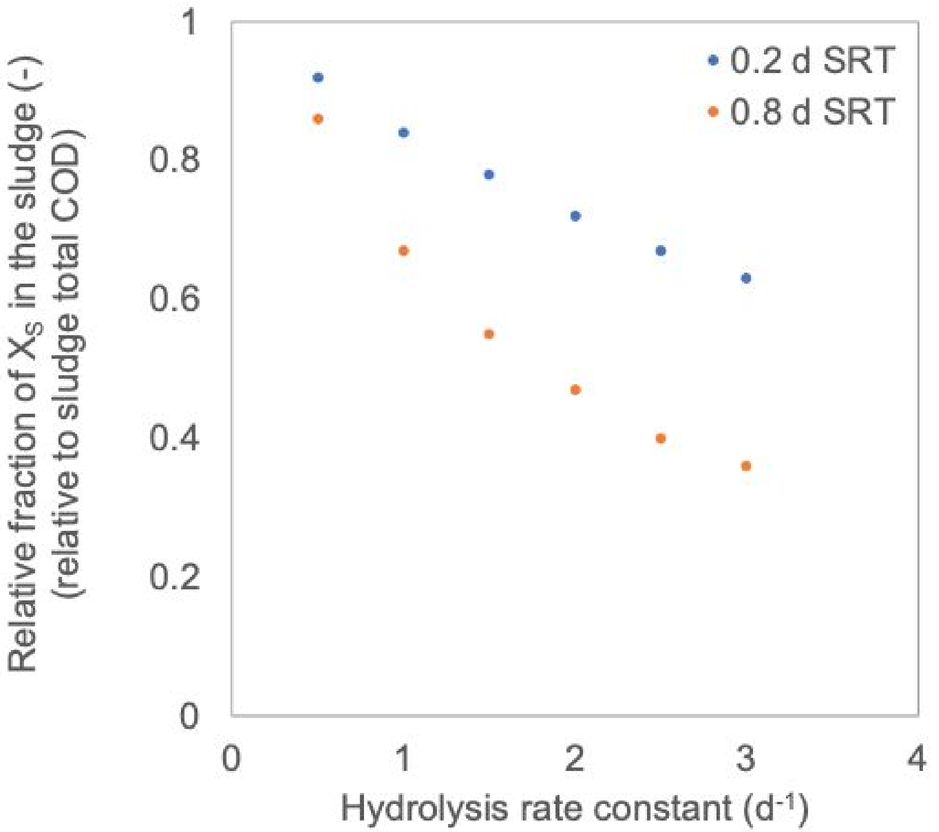

Figure 1: Change in the fraction of Xs (-) as a function of the hydrolysis rate constant (d^{-1}).

Reference

Henze, M., W. Gujer, T. Mino and M. van Loosdrecht (2000). Activated sludge models ASM1, ASM2, ASM2d and ASM3. Edited by IWA task group on mathematical modelling for design and operation of biological wastewater treatment. IWA Scientific and Technical Report. ISBN: 1 900222 24 8. ISSN: 1025-0913.
